# Photoperiod, light intensity, and vernalization regulate flowering in the novel fragrant Chrysanthemum ‘Xiaokuixiang’

**DOI:** 10.3389/fpls.2026.1865852

**Published:** 2026-07-01

**Authors:** Yujing Deng, Youlituzi Alimu, Jiahua Li, Ruichen Tian, Miaomiao Song, Zhengyi Wang, Yixiang Wang, Palinuer Aiwaili

**Affiliations:** 1 College of Horticulture, Xinjiang Agricultural University, Ürümqi, China; 2 College of Horticulture, China Agricultural University, Beijing, China

**Keywords:** *Chrysanthemum morifolium* ‘Xiaokuixiang’, flowering regulation, light intensity, photoperiod, vernalization

## Abstract

Accurate regulation of the flowering period is crucial for year-round production of Chrysanthemum. This study was conducted in an artificial climate chamber to systematically investigate the effects of light intensity, photoperiod, and vernalization on the growth and flowering of a new fragrant Chrysanthemum (*Chrysanthemum morifolium* ‘Xiaokuixiang’). Results showed that ‘Xiaokuixiang’ is a facultative short-day plant requiring at least 12 hours of darkness for flower bud differentiation, with 16 hours of darkness further optimizing this process. High light intensity (300 μmol m^-^² s^-^¹) significantly accelerated both vegetative growth and flowering. Although vernalization did not directly alter the differentiation rate under short-day conditions, it effectively shortened the vegetative growth phase and increased the number of normal flower buds. Combined treatment analysis revealed that plants pre-treated with low temperature exhibited optimal flowering performance under 300 μmol m^-^² s^-^¹ light intensity and short-day conditions (8 hours of light/16 hours of darkness). Based on these findings, an optimized production strategy is proposed: using winter-cold-exposed basal shoots as cutting materials, providing sufficient light during the vegetative stage, and promptly initiating short-day induction to achieve precise control over the flowering period of ‘Xiaokuixiang’.

## 1 Introduction

The cultivated chrysanthemum(*Chrysanthemum morifolium* Ramat.), recognized as one of the four major cut flower species globally, boasts a long-standing cultivation history and holds remarkably high economic and cultural significance across the world (Huang et al., 2026). In recent years, with the diversification of consumer markets, tea chrysanthemum, which integrates ornamental, medicinal, and beverage value, has gained significant attention. This is partially attributed to the anti-inflammatory and antioxidant activities of vesicles derived from food-medicine homologous plants (Shu et al., 2025, 2024). However, the year-round production of chrysanthemums heavily relies on precise flowering regulation techniques. This not only affects the continuous supply of products but also directly influences their commercial quality and production costs (Liu et al., 2024). Floral induction in chrysanthemums is a complex physiological process governed by the intricate integration of environmental cues, primarily photoperiod, light intensity, and temperature. While the independent effects of these factors are documented, the synergistic mechanisms regulating the transition from vegetative to reproductive growth—especially in novel cultivars with unique aromatic traits—remain insufficiently understood.

Floral induction in plants is a complex physiological process coordinately regulated by a multitude of internal and external signals. Plants utilize five major flowering pathways, namely the photoperiod pathway, vernalization pathway, gibberellin pathway, autonomous pathway, and age-dependent developmental pathway (Mouradov et al., 2002; Boss et al., 2004). Among external environmental factors, light and temperature are core signals that regulate the transition from vegetative growth to reproductive growth in plants (Zhou et al., 2018). The flowering signals from these pathways are ultimately integrated into floral regulators including *FT*, *SOC1*, and the floral integrator *LEAFY* (*LFY*), thereby forming a complex gene regulatory network (Yamaguchi and Abe, 2012).

Light intensity plays a critical role in the flowering process, exerting its effects by modulating the carbon assimilation capacity and energy accumulation of plants (Huang, 2014). Under appropriate light intensity, the photosynthetic and transpiration rates of plants are enhanced, which in turn boosts their biomass production capacity and dry matter accumulation, thereby facilitating plant growth and development (Torres and Lopez, 2011). Light intensity also exerts a significant influence on the flowering of ornamental plants. Studies have demonstrated that as light intensity increases, the time required for visible flower bud formation and anthesis in roses is shortened (Hopper and Hammer, 1991). Photoperiod is a key determinant of chrysanthemum flowering. Most autumn-flowering chrysanthemum cultivars are typical short-day plants (SDPs), with a critical photoperiod typically ranging from 10 to 13 hours, and the exact duration varies by cultivar (Lu and Yang, 2021). Different chrysanthemum varieties exhibit distinct sensitivities to day length. For instance, in summer-flowering chrysanthemums, the balance between vegetative and reproductive growth is strictly regulated by B-box proteins such as CmBBX5 and CmBBX8, which prevents premature flowering (Wang et al., 2024).

Beyond photoperiod, low temperature can induce flowering in certain plant species. Vernalization refers to the physiological process by which plants require prolonged cold exposure to trigger the transition from vegetative to reproductive growth. Vernalization not only necessitates plants to perceive cold stress but also involves the formation of a “memory” of this stress, ensuring their ability to respond and flower upon warming in spring (Xu and Chong, 2018). Vernalization serves as a necessary prerequisite or promoting factor for flowering in many Asteraceae plants. Appropriate low-temperature treatment can induce the expression of flowering-related genes, thereby facilitating floral bud differentiation (Lyu et al., 2022).

At the molecular level, *CmFTL3* in chrysanthemums acts as a core component of florigen and plays a pivotal role in integrating environmental signals and triggering the transition of the shoot apical meristem (*SAM*) to a floral meristem (Kobayashi and Weigel, 2007; Takahashi et al., 2025). In the research on chrysanthemum, under short-day conditions, *CsFTL3* induces the expression of the flower organ identity gene *CsAFL1* in leaves, but it fails to do so under long-day conditions (Nakano et al., 2019). Temperature signals, particularly vernalization, are typically mediated by repressor factors such as *FLC* (Flowering Locus C), and the downregulation of these repressors is a prerequisite for flowering initiation (Xu and Chong, 2018; Tao et al., 2017; Andrés and Coupland, 2012). While the molecular functions of *CmFTL3* and *FLC* have been elucidated, a critical knowledge gap persists regarding how their expression dynamics are coordinated under the combined stress of specific light intensities and cold exposure durations in novel germplasm materials.

Chrysanthemums exhibit diverse flowering ecotypes owing to their extensive geographical distribution, with distinct ecotypes displaying unique environmental dependencies on the key factors that trigger flowering. ‘Xiaokuixiang’ is a novel fragrant chrysanthemum cultivar developed by our research team, which integrates high ornamental value with edible potential (Wang et al., 2021). However, the specific environmental thresholds required for flowering induction in this cultivar remain uncharacterized. Therefore, this study employed ‘Xiaokuixiang’ as the experimental material to systematically investigate the effects of varying light intensities, photoperiod treatments, and low-temperature induction durations on plant morphology, flowering progression, and floral quality. It is important to note that the limitations of this study lie in its exclusive focus on phenotypic responses and the expression of key flowering genes under controlled environmental conditions, without exploring upstream signaling transduction networks or proteomic alterations in depth.

Accordingly, we hypothesize that specific high light intensity, appropriate photoperiod, and proper vernalization can significantly reduce the flowering duration of ‘Xiaokuixiang’ and enhance floral quality. To test this hypothesis, the specific objectives of this study are as follows: (1) To clarify the individual and interactive effects of light intensity, photoperiod, and low-temperature induction on the morphological traits and flowering time of ‘Xiaokuixiang’; (2) To analyze the expression patterns of flowering-related genes under the aforementioned treatments; (3) To establish an optimal cultivation protocol for the precise regulation of flowering in this new cultivar.

## 2 Materials and methods

### 2.1 Plant materials and cultivation

This study employed the pear-scented chrysanthemum cultivar *Chrysanthemum morifolium* cv. ‘Xiaokuixiang’, bred by China Agricultural University. Plant materials for light intensity and photoperiod treatment experiments were obtained via cutting propagation: 5 cm-long cuttings were rooted in a solar greenhouse. The plant materials used in the low-temperature treatment experiment were derived from tissue culture. This is because tissue-cultured plants, which have not been exposed to natural low-temperature conditions and exhibit uniform growth status, satisfy the single-variable requirement for low-temperature treatment experiments. The culture medium used was ½ MS medium, with pH adjusted to 5.8–6.0. When cuttings reached 10 cm in height and tissue-cultured seedlings grew to 6 cm, uniformly developed plants were selected and transplanted into 8 cm × 8 cm pots. The potting substrate consisted of a 1:1 (v/v) mixture of vermiculite (Hebei Yixiang Vermiculite Co., Ltd., particle size 1–3 mm) and peat moss (Pindstrup Mosebrug A/S, Denmark, particle size 0–10 mm), which had been autoclaved at 121 °C for 30 min. The electrical conductivity (EC) of the final mixed substrate, determined via saturated extraction followed by conductivity meter measurement, was 0.8–1.2 mS·m^-^¹. Subsequently, plants were cultivated in artificial climate chambers under LD (long-day 16 h light/8 h dark) and SD (short-day 8 h light/16 h dark) conditions, with a temperature of 22–24 °C and relative humidity of 40%.

### 2.2 Experimental apparatus

A full-spectrum LED light source (Guangdong Weizhaoye Optoelectronic Energy Saving Co., Ltd., Zhongshan, China) was employed in this experiment. Derived from conventional LED lamps, this full-spectrum LED lamp incorporates additional red light, which better facilitates plant growth and flowering. The spectral distribution is presented in **Supplementary Figure 1** of the **Supplementary Materials**. To ensure the apical meristems of the plants were exposed to a consistent light environment throughout the experiment, the distance between the plant apical meristems and the light source was maintained at 15–20 cm at all times. A illuminance meter (Delixi Electric DLY-1802, China) was used to measure light intensity at multiple positions (corners and center) within the experimental area, with a coefficient of variation (CV) of <5%, confirming satisfactory light uniformity.

### 2.3 Treatment with different light intensities

Cuttings of ‘Xiaokuixiang’ (each treatment consisted of 30 plants, with 5 randomly selected for molecular analysis, each plant considered an individual biological replicate.) were cultivated at 21-23 °C. Four light intensity gradients were established: (1) 300 μmol m^-^² s^-^¹; (2) 200 μmol m^-^² s^-^¹; (3) 100 μmol m^-^² s^-^¹; (4) 50 μmol m^-^² s^-^¹. Sampling was conducted at ZT3 (3 hours after lights on) on day 16 of short-day (SD) treatment. The apical buds and the fourth leaf from the apex were collected, immediately flash-frozen in liquid nitrogen, and subsequently stored at -80 °C.

### 2.4 Treatment with different photoperiods

Cuttings of ‘Xiaokui Xiang’ were subjected to 45 days of vegetative growth under long-day (LD) conditions at 21–23 °C with a light-dark cycle of 16/8 hours and a light intensity of 300 μmol m^-^² s^-^¹. Subsequently, the plants (each treatment consisted of 30 plants, with 5 randomly selected for molecular analysis, each plant considered an individual biological replicate.) were transferred into four different photoperiod treatments: (1) 8/16 hour light-dark cycle; (2) 10/14 hour light-dark cycle; (3) 12/12 hour light-dark cycle; and (4) 16/8 hour light-dark cycle.

Normal flower buds were collected two weeks after the start of short-day treatment (when the axillary terminal buds began to differentiate into flower buds). Abnormal flower buds were collected at three stages: (1) one month after transplantation (under long-day conditions) when they first appeared, with a flower bud diameter of 1 mm; (2) one week after being moved to short-day conditions, during the period of continued abnormal flower bud development; (3) three weeks after the appearance of abnormal flower buds under continuous long-day conditions, when the flower bud diameter reached 3 mm. All collected flower buds were rapidly frozen in liquid nitrogen and stored at -80 °C.

### 2.5 Low-temperature treatments of different durations

Tissue-cultured seedlings that had been cultured *in vitro* for 30 days (growing to approximately 6 cm in height) were first rinsed thoroughly to remove residual medium from their roots. The seedlings were then subjected to acclimatization for 3 days under long-day conditions (16 h light/8 h dark) at 22 °C–24 °C, with their roots immersed in clean water. Following acclimatization, the seedlings were transplanted under the conditions specified in Section 2.1 of Materials and Methods, The seedling survival rate is 100%. Treatments were initiated when the plants reached approximately 10 cm in height under normal temperature conditions. The experiment comprised six low-temperature treatment durations: plants grown at normal temperature served as the control group (LT0), while the low-temperature groups (LT1-LT5) were subjected to 4 ± 1 °C under long-day (LD, 16 h light/8 h dark) conditions for LT1 (1 week), LT2 (2 weeks), LT3 (3 weeks), LT4 (4 weeks), and LT5 (5 weeks), respectively. Upon completion of the treatments, all groups were transferred to a normal temperature (22 °C-24 °C) long-day environment for continued growth. When the plant height of the LT5 group reached 26 cm, the photoperiod was uniformly adjusted to short-day (SD, 8 h light/16 h dark) conditions for all plants.

During the low-temperature treatment period, samples were collected weekly from the low-temperature treatment groups (LT1-LT5) and their corresponding normal-temperature control groups (C1-C5). After the low-temperature treatment concluded, the LT5 group was sampled again at 1 week (LT5 + 1 w) and 5 weeks (LT5 + 5 w) of recovery growth. The sampling site was the fourth leaf from the plant apex. Samples were immediately frozen in liquid nitrogen and stored at -80 °C. Five biological replicates were included for each sampling time point.

### 2.6 Stereomicroscopy and scanning electron microscopy observation

The involucral bracts of the plants were carefully dissected under a stereomicroscope (Nikon SMZ800) to expose the apical meristem. Subsequently, the exposed apical meristem was observed and photographed using a scanning electron microscope (Hitachi TM3000 Tabletop SEM).

### 2.7 RNA extraction and qRT-PCR analysis

Total RNA was extracted using the TRIzol method, and its concentration and purity were assessed with a NanoDrop 2000 spectrophotometer (Thermo Fisher Scientific, USA). Subsequently, 2 μg of total RNA was used for genomic DNA removal and reverse transcription into cDNA using the HiScript II Q Select RT SuperMix for qPCR (+gDNA wiper) kit (Vazyme, China).qRT-PCR was performed on a StepOne real-time PCR system (Applied Biosystems, USA) using the KAPA SYBR FAST qPCR kit (Kapa Biosystems, USA) to detect the expression levels of the *CmFTL3*, *CmSOC1*, *CmGA20ox1*, *CmCOL1*, and *CmFLC* genes. *CmUBI* (GenBank accession NM_112764) was used as the internal reference gene (Czechowski et al., 2005), and relative expression levels were calculated using the 2^-ΔΔCt^ method (Livak and Schmittgen, 2001). The gene-specific primers are listed in **Supplementary Table 1**. The thermal cycling program was set as follows: 95 °C for 10 min; 40 cycles of 94 °C for 20 s and 60 °C for 30 s; followed by a melting curve analysis from 65 °C to 95 °C. Five biological replicates were included for each sample.

### 2.8 Data processing

All experiments included at least three biological replicates. One-way analysis of variance (ANOVA) with the least significant difference (LSD) method was employed, and the significance level was set at P < 0.05. Subsequently, charts and graphs were generated using GraphPad Prism v8.0 (GraphPad Software, San Diego, CA, USA).

## 3 Results

### 3.1 Light intensity primarily promotes early flowering by accelerating vegetative growth

To investigate the effects of different light intensities on the vegetative growth of chrysanthemum, all treatment groups were cultivated under long-day (LD) conditions for 45 days to ensure sufficient vegetative growth. Subsequently, they were transferred to short-day (SD) conditions for continued cultivation, and the growth status of plants in each treatment group was continuously observed. After the transfer to SD conditions, significant morphological differences and variations in flowering time were observed among the treatment groups.

Observations revealed that the chrysanthemum cultivar ‘Xiaokuixiang’ could initiate floral bud differentiation under LD conditions. Following adequate vegetative growth, willow leaf-shaped leaves and flower buds enveloped by willow leaf-shaped bracts appeared at the apical bud. However, these buds ceased development upon emergence. To distinguish between the buds that abort under LD conditions and those that develop normally, the former are referred to as developmentally arrested flower buds in this study. The emergence of developmentally arrested flower buds disrupts apical dominance, promoting the occurrence of lateral buds. Subsequently, the newly emerged lateral buds undergo floral initiation but arrest development prior to full morphological differentiation. Therefore, the appearance of developmentally arrested flower buds under LD conditions is considered a key node, signaling the transition from vegetative to reproductive growth in chrysanthemum.

**Figure 1:**
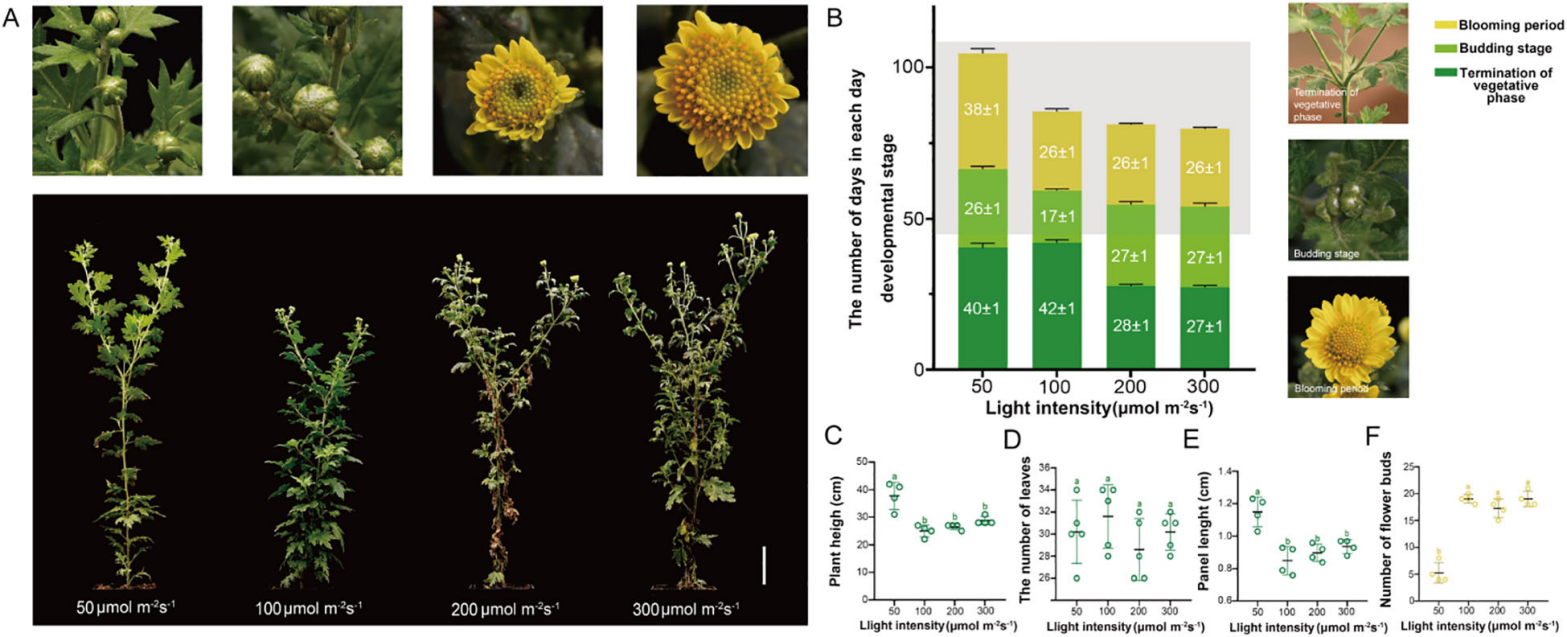
Phenotypic characteristics of ‘Xiaokuixiang’ under different light intensities. **(A)** Flowering phenotype; Scale bar = 5 cm. **(B)** Growth period, the shaded area indicating the start of short-day conditions. **(C)** Plant height. **(D)** Leaf number. **(E)** Internode length. **(F)** Number of flower buds.

Following treatment with different light intensities, the 200 μmol m^-^² s^-^¹ and 300 μmol m^-^² s^-^¹ treatment groups flowered earliest, followed by the 100 μmol m^-^² s^-^¹ group. The 50 μmol m^-^² s^-^¹ treatment group exhibited the latest bud appearance (**Figure 1A**). Floral bud initiation occurred earliest in the 200 μmol m^-^² s^-^¹ and 300 μmol m^-^² s^-^¹ treatment groups, with average initiation times of 28 ± 1 days and 27 ± 1 days after planting, respectively. The initiation times for the 50 μmol m^-^² s^-^¹ and 100 μmol m^-^² s^-^¹ groups were significantly later than the former two groups, occurring at 40 ± 2 days and 42 ± 1 days after planting, respectively. After transfer to SD conditions, the 200 μmol m^-^² s^-^¹ and 300 μmol m^-^² s^-^¹ groups completed floral bud differentiation and flowering first, followed by the 100 μmol m^-^² s^-^¹ group. The 50 μmol m^-^² s^-^¹ group flowered latest (**Figure 1B**).

Plant height and internode length differed significantly among treatment groups. The 50 μmol m^-^² s^-^¹ group exhibited significantly greater plant height and internode length than the other three groups (100, 200, 300 μmol m^-^² s^-^¹), while no significant differences were observed among the latter three groups(**Figures 1C–E**). Statistical analysis of flower bud numbers revealed that the 50 μmol m^-^² s^-^¹ group had a significantly lower number of flower buds compared to the other groups. However, the final flower size and morphology were relatively consistent across all groups (**Figure 1F**). These results collectively indicate that, to a certain extent, light intensity affects the normal development of chrysanthemum flower buds, manifested as fewer normally opening flower buds under lower light intensities. Nevertheless, the morphology of individual flowers after anthesis was not influenced by light intensity. In summary, under suitable light conditions, increased light intensity facilitates vegetative growth and promotes earlier floral bud initiation.

### 3.2 Analysis of key flowering gene expression under different light intensities

**Figure 2:**
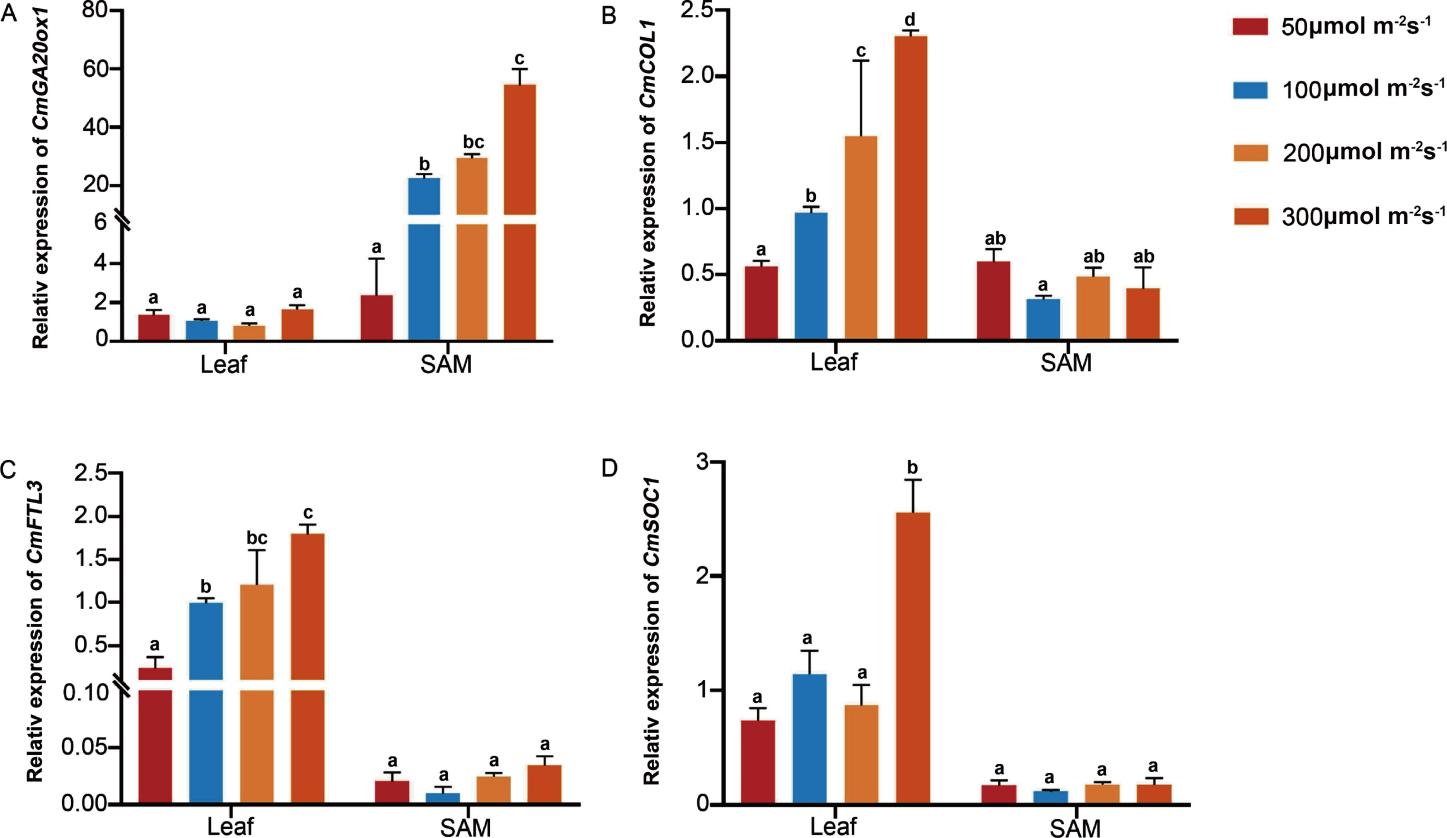
Expression analysis of flowering-related genes in ‘Xiaokuixiang’ under different light treatments **(A–D)**. Relative expression of *CmGA20ox1* **(A)**. *CmCOL1* **(B)**. *CmFTL3* **(C)**. *CmSOC1* **(D)**. SAM, shoot apical meristem.

To further investigate the effects of light intensity on floral induction in chrysanthemum at the genetic level, the expression levels of genes related to chrysanthemum floral induction were examined. In chrysanthemum, GA oxidases are key enzymes in the synthesis and degradation of bioactive GAs, controlling endogenous GA levels, among which *CmGA20ox* is an oxidase responsible for synthesizing bioactive GAs. As shown in **Figure 2A**, the expression level of the *CmGA20ox1* gene in the apical buds was significantly higher than that in the leaves. Within the apical buds, the expression was highest in the 300 μmol m^-^² s^-^¹ treatment group, followed by the 200 μmol m^-^² s^-^¹ and 100 μmol m^-^² s^-^¹ treatment groups, whose expression levels were approximately 0.5-fold that of the 300 μmol m^-^² s^-^¹ group. The lowest expression was observed in the 50 μmol m^-^² s^-^¹ treatment group. This overall expression trend corresponded with the bud emergence times described above. As a core transcription factor in the photoperiod pathway, the expression pattern of *CmCOL1* in the leaves was similar to that of the florigen gene *CmFTL3*, both being significantly down-regulated with decreasing light intensity and exhibiting specific expression in the leaves (**Figures 2B, C**). Among them, the *CmFTL3* expression level was highest in the 300 μmol m^-^² s^-^¹ treatment group and lowest in the 50 μmol m^-^² s^-^¹ group. Similarly, the expression level of the flowering integrator gene *CmSOC1* was higher in the leaves than in the apical buds (**Figure 2D**). In the leaves, the 300 μmol m^-^² s^-^¹ treatment significantly induced high expression of *CmSOC1*, while the differences in expression among the other low-light intensity groups were not significant.

These results indicate that *CmGA20ox1*, *CmCOL1*, *CmFTL3*, and *CmSOC1* are involved in regulating the floral transition of ‘Xiaokuixiang’, and their expression is induced by light intensity. Under higher light intensities, the expression of these genes is up-regulated, associated with promoting flowering in ‘Xiaokuixiang’. Conversely, under lower light intensities, expression is suppressed, associated with delayed flowering.

### 3.3 Photoperiod is the dominant environmental factor controlling floral transition

To investigate the effects of different photoperiod treatments on the flowering time of ‘Xiaokuixiang’, all treatment groups were cultivated under the optimal light intensity condition determined from the light intensity experiment, namely 300 μmol m^-^² s^-^¹. They were initially grown under long-day (LD, 16 h light/8 h dark) conditions for 45 days to ensure sufficient vegetative growth before being transferred to different photoperiod regimes. The growth status of the apical buds in each treatment group was continuously observed.

**Figure 3:**
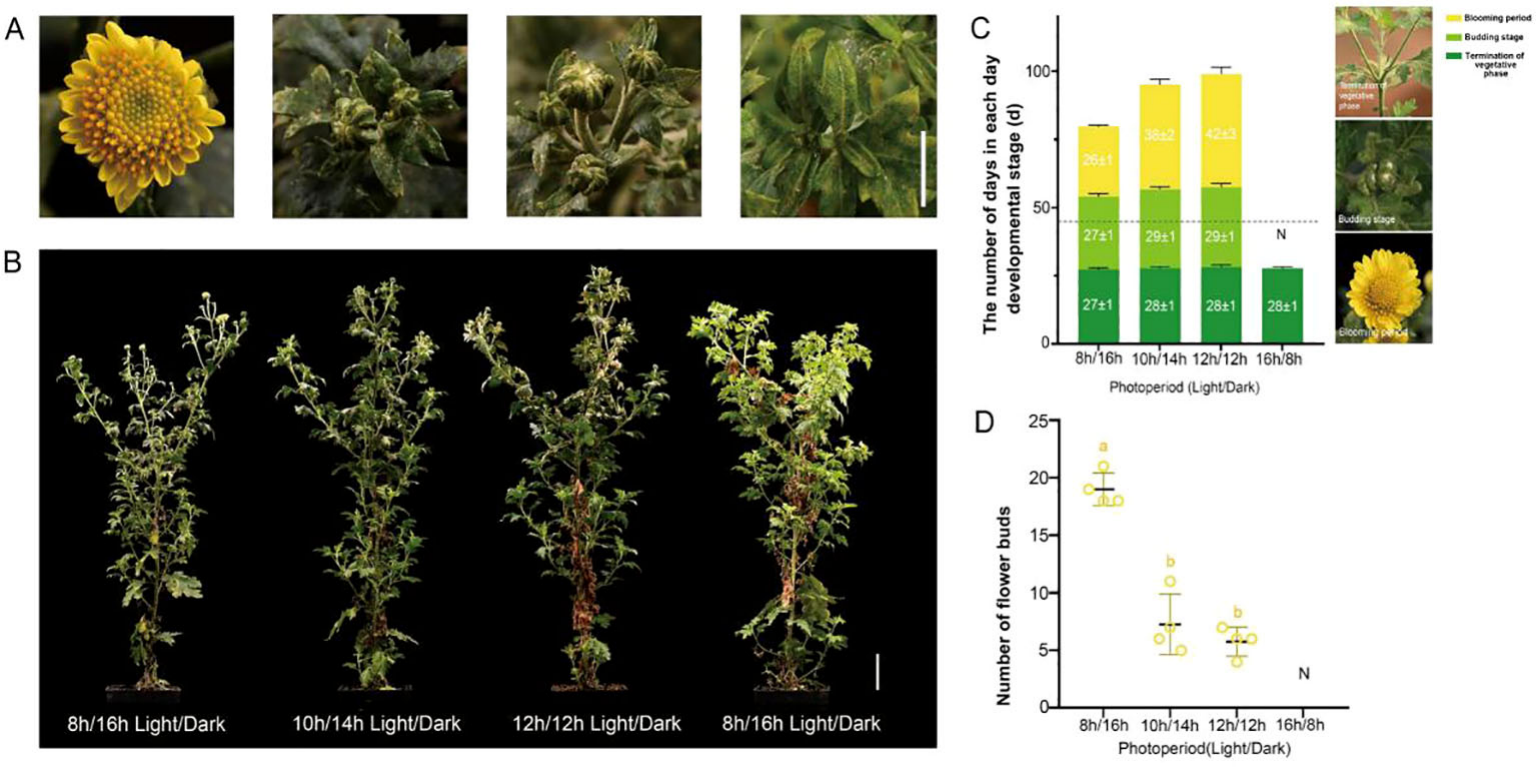
Phenotypic characteristics of ‘Xiaokuixiang’ under different photoperiods. **(A,B)** Flowering phenotypes **(A)** and growth morphology **(B)**, scale bar = 1 cm **(A)** and 5 cm **(B)**. **(C)** Growth stage; dotted lines indicate the start of the transition from long-day treatment to different photoperiod treatments. **(D)** Number of normally opening flower buds.

Following the different photoperiod treatments and statistical analysis of each flowering stage, it was found that under LD conditions, flower buds remained in a malformed state, continuously producing abnormal buds that failed to develop normally. Neutral-day (ND, 12 h light/12 h dark) conditions allowed for normal bud emergence and flowering, while short-day (SD) conditions accelerated the flowering process as the light duration decreased (**Figures 3A, B**). The 8 h/16 h (SD) treatment group completed floral differentiation and initiated bud emergence earliest, with an average bud emergence time of 54 ± 1 days. The average bud emergence times for the 10 h/14 h and 12 h/12 h treatment groups were later than that of the 8 h/16 h group, at 57 ± 1 days. The 16 h/8 h (LD) treatment group ultimately failed to produce any normally developing flower buds. As bud development progressed, the 8 h/16 h group flowered earliest, with an average duration from bud emergence to anthesis of 26 ± 1 days. The average durations for the 10 h/14 h and 12 h/12 h groups were 38 ± 2 days and 42 ± 3 days, respectively. The malformed buds in the 16 h/8 h treatment could not develop further (**Figure 3C**). Statistical analysis of the number of normally opened flowers in each treatment group revealed that, as shown in **Figure 3D**, the number of flowers capable of normal anthesis decreased with increasing day length. In the 10 h/14 h and 12 h/12 h treatment groups, some buds failed to open completely due to photoperiod influence. Under LD (16 h/8 h) conditions, plants continuously produced lateral branches without flowering, consistently affected by malformed buds. Collectively, these results indicate that photoperiod affects the normal development of chrysanthemum flower buds and is the dominant environmental factor controlling floral transition in ‘Xiaokuixiang’.

### 3.4 Developmental patterns of developmentally arrested flower buds and expression profiles of key genes

Since developmentally arrested flower buds appear but fail to develop normally under long-day conditions, scanning electron microscopy (SEM) was employed to investigate the micro-morphological differences between malformed and normal flower buds.

**Figure 4:**
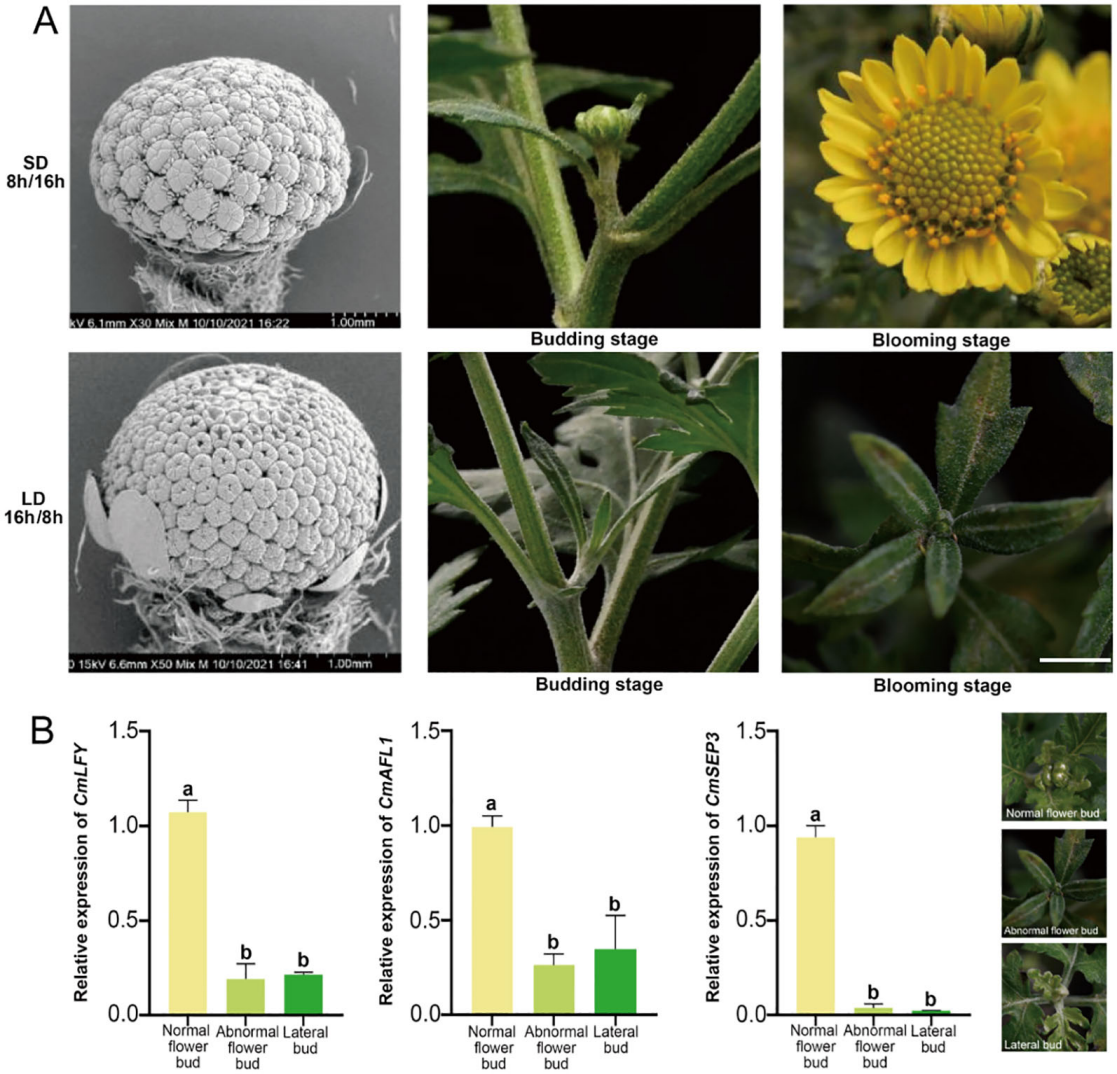
Developmentally arrested flower buds phenotype. **(A)** Developmentally arrested flower buds under short-day (SD) long-day (LD) and treatment. scale bar = 1 cm. **(B)** Expression of flowering-related genes in flower buds at different developmental stages.

SEM observations revealed that the developmentally arrested flower buds formed under long-day conditions exhibited a high degree of morphological similarity to normal flower buds induced under short-day conditions during the early developmental stages (**Figure 4A**). Both had completed the floral transition and differentiated all bract and floret primordia, indicating that ‘Xiaokuixiang’ retains the capacity to initiate floral bud differentiation under long-day conditions.

To investigate the developmental fate of developmentally arrested flower buds under different photoperiods, the plants bearing malformed buds were subjected to either short-day (8 h light/16 h dark) or long-day (16 h light/8 h dark) treatments after the buds appeared. As shown in **Figure 4A**, developmentally arrested flower buds under short-day treatment completed floral differentiation and continued to develop, exhibiting morphology and developmental processes similar to normal buds, and eventually opened normally. In contrast, under long-day treatment, the malformed buds did not develop further and gradually withered over time. Subsequently, new developmentally arrested flower buds emerged from the axillary meristems below the original buds, and this cycle repeated. None of the malformed buds under long-day conditions opened normally.

To further determine whether malformed buds represent normal floral structures, the expression levels of key genes involved in the initiation and development of chrysanthemum floral organs were analyzed in developmentally arrested flower buds, enlarged axillary buds from the shoot apex, and normal floral buds (**Figure 4B**). The results showed that although the malformed buds had morphologically completed the initial floral transition and formed floret primordia, the expression of *CmLFY*, a key gene regulating floral meristem identity, was significantly lower than in normally developing buds. Furthermore, the expression of *CmAFL1* and *CmSEP3*, key genes in the ABCE model regulating floral organ development, was significantly lower in malformed buds and axillary buds compared to normal buds. These findings indicate that under long-day conditions, while plants can initiate the floral transition, the transcriptional activity of genes associated with floral meristem and floral organ development is environmentally suppressed. This inhibition prevents the further development and elongation of floret primordia, ultimately leading to the formation of developmentally arrested, developmentally arrested flower buds.

### 3.5 Expression analysis of key flowering genes under different photoperiods

**Figure 5:**
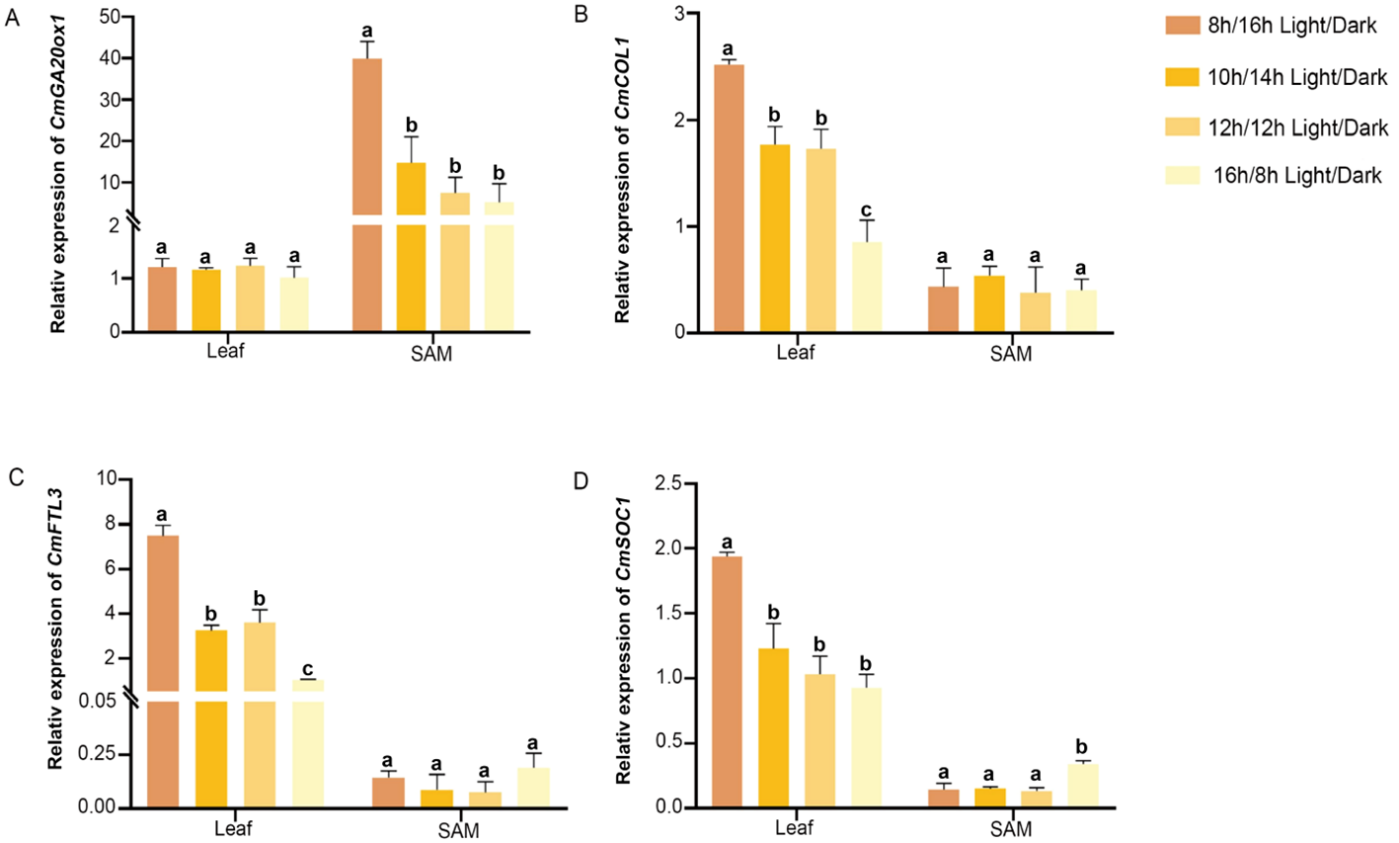
Expression analysis of flowering-related genes in different organs of ‘Xiaokuixiang’ under different photoperiod treatments. **(A–D)** Relative expression of *CmGA20ox1* **(A)**. *CmCOL1* **(B)**. *CmFTL3* **(C)**. *CmSOC1* **(D)**. SAM, shoot apical meristem.

To further elucidate the photoperiodic regulation of flowering in ‘Xiaokuixiang’, this study analyzed the expression patterns of key flowering-related genes under different day lengths (**Figure 5A**). Similarly, the expression level of the *CmGA20ox1* gene in apical buds was significantly higher than that in leaves and exhibited a distinct short-day induction characteristic: its expression was highest under the 8 h light/16 h dark (8L/16D) treatment and lowest under the 16 h light/8 h dark (16L/8D) treatment. In contrast, the expression levels of the *CmCOL1*, *CmFTL3*, and *CmSOC1* genes were higher in leaves than in apical buds. However, their response trends to photoperiod were consistent with that of *CmGA20ox1*, showing significant downregulation with increasing day length (**Figures 5B–D**). The overall expression patterns of these genes were highly correlated with the bud emergence times observed across treatment groups. The highest gene expression was detected in the treatment group with the shortest day length, while the lowest expression was observed under long-day conditions. These results indicate that short-day conditions accelerate the floral transition in ‘Xiaokuixiang’ by synergistically upregulating the expression of key genes in the flowering pathway.

### 3.6 Effects of low-temperature on the plant architecture of ‘Xiaokuixiang’

**Figure 6:**
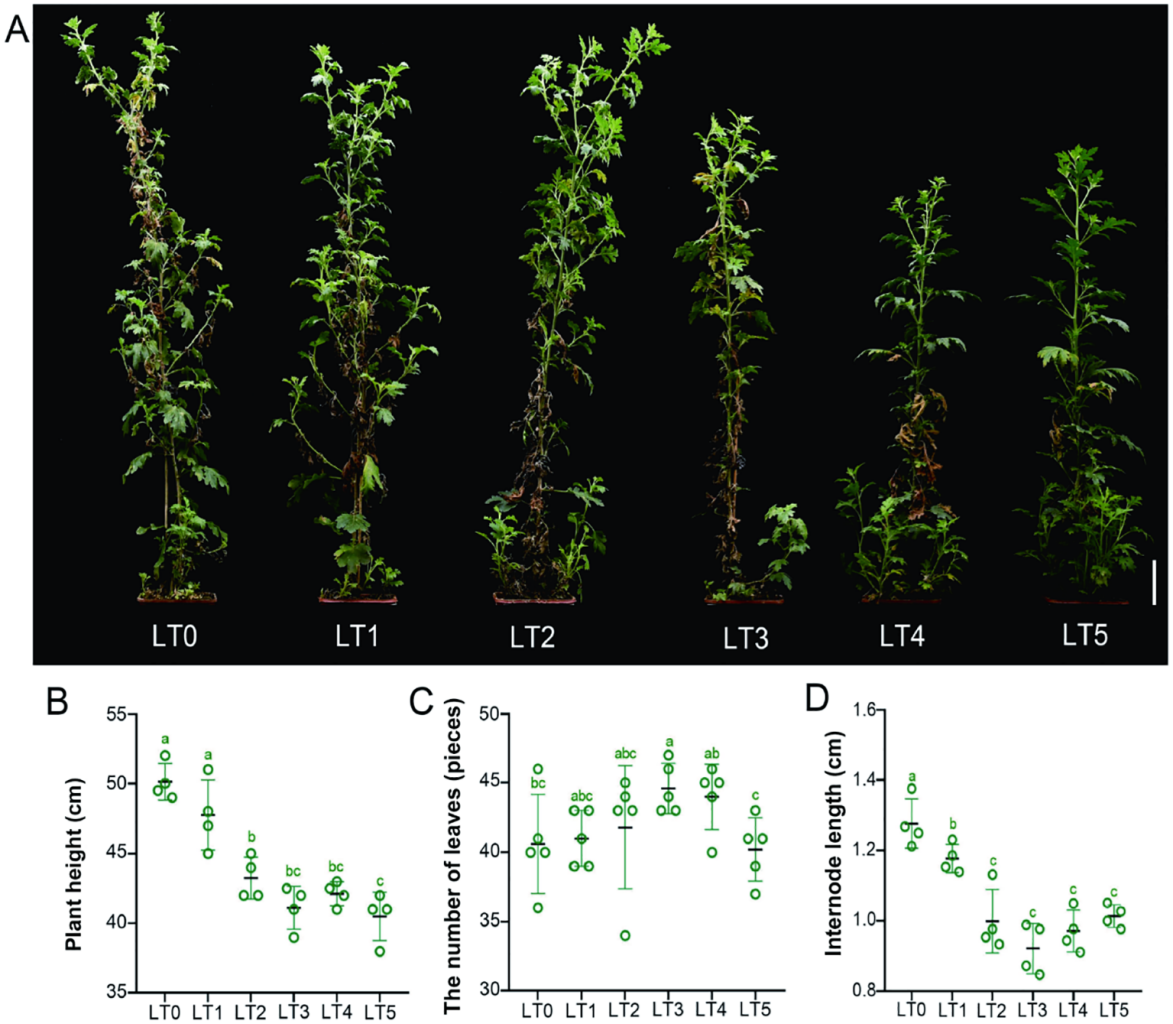
Phenotypes of ‘Xiaokuixiang’ after low-temperature treatments of varying durations. **(A)** Growth morphology, scale bar = 5 cm. **(B–D)** Plant height **(B)**. Leaf number **(C)** and internode lengths **(D)** under different treatments. LT, low temperature.

To investigate whether low-temperature treatments of varying durations affect the flowering time of ‘Xiaokuixiang’, plants subjected to gradient low-temperature treatments of 1 to 5 weeks (LT1-LT5) were transferred to a normal temperature environment after the treatment concluded. Plants continuously grown at normal temperature (LT0) served as the control. The growth status of each treatment group was monitored continuously. As shown in **Figures 6A, B**, the LT0 group, which did not undergo low-temperature treatment, exhibited the greatest plant height among all groups, followed by the LT1 group. The plant heights of the LT4 and LT5 groups were relatively shorter compared to other treatments. Plant height decreased progressively with increasing duration of low-temperature exposure. Statistical analysis of plant height and internode length for each group (**Figures 6C, D**) revealed a gradient change in plant height, which diminished as the low-temperature treatment duration increased. Since the number of leaves showed no significant differences among groups, the internode length, similar to plant height, shortened with prolonged low-temperature treatment. Consequently, plants from the LT4 and LT5 treatment groups displayed a more compact architecture. In summary, the differences in growth status among the treatment groups were manifested in plant height and internode length, with no significant variation observed in leaf number.

### 3.7 Low-temperature does not significantly affect flowering time but increases bud number

**Figure 7:**
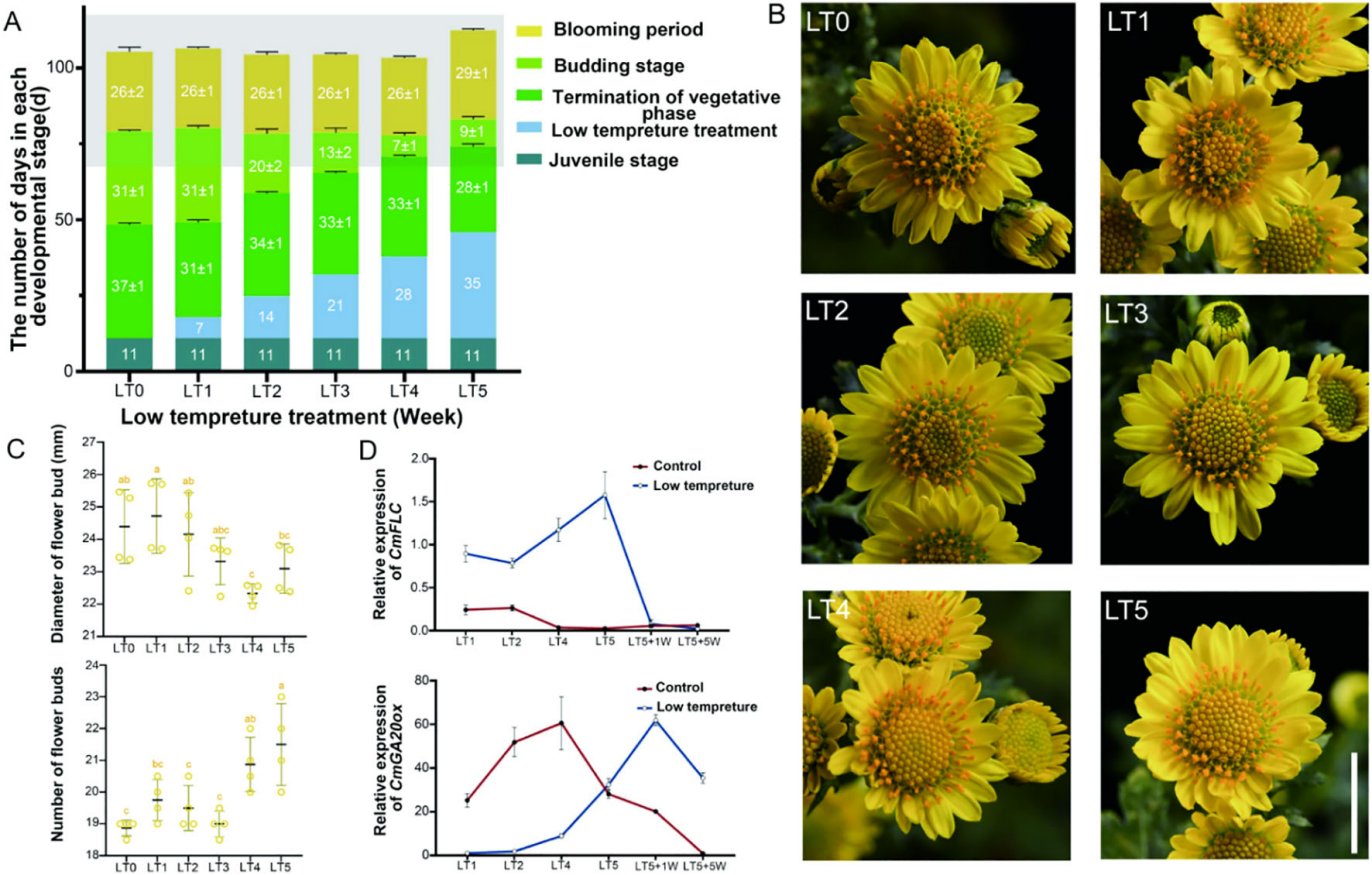
Flowering phenotypes in ‘xiaokuixiang’ following low-temperature treatments of varying durations. **(A)** Growth periods under different treatment with the shaded area indicating the start of short-day conditions. **(B)** Inflorescence opening morphology. scale bar = 1 cm. **(C)** Inflorescences diameter and number. **(D)** Expression of flowering-related genes. LT, low temperature.

To investigate whether low-temperature treatments of varying durations affect the flowering time of chrysanthemums, the developmental status of apical buds in each treatment group was monitored continuously. In the prior light intensity experiment, plants in the 300 μmol m^-^² s^-^¹ treatment group completed vegetative growth when their height reached 28 ± 1 cm. Thus, when plants in the LT5 treatment group grew to approximately 28 cm under normal temperature conditions, all treatment groups were transferred to short-day (SD) conditions for floral bud induction. Observations and statistical analyses of plant growth at different stages revealed that low-temperature treatment significantly altered the progression of ‘Xiaokuixiang’ from transplanting to the reproductive growth phase. Notably, the two most prominently affected stages were the termination of the vegetative phase and the budding stage: as the duration of low-temperature treatment increased, the total time from transplanting to the emergence of the first floral bud prolonged gradually (from 48 ± 1 days in the LT0 group to 74 ± 1 days in the LT5 group). However, further analysis indicated that the actual vegetative growth period under normal temperature conditions was significantly shortened with increasing low-temperature treatment duration: the LT0 group required 37 ± 1 days, whereas the LT5 group needed only 28 ± 1 days. This suggests that low-temperature treatment effectively accelerates internal floral transition and reduces the time required for vegetative growth under optimal temperature conditions (**Figure 7A**).

Regarding the developmental pattern, the apices of all low-temperature-treated plants first formed terminal floral buds, followed by the development of three lateral branches, on which normal floral buds differentiated. After transfer to SD conditions for induction, a high degree of developmental synchrony was observed across all treatment groups. Statistical analysis showed no significant differences in the time from SD induction to floral bud emergence among the groups (11 ± 1 days for LT1–LT4 and 14 ± 1 days for LT5). Similarly, the duration from floral bud emergence to inflorescence opening was largely consistent across groups, ranging from 26 to 29 days (**Figure 7A**). These results indicate that low-temperature treatment primarily regulates flowering indirectly by shortening the vegetative growth period under normal temperatures. Once plants enter the SD induction phase, low-temperature has no significant effect on the rate of floral bud differentiation or inflorescence opening.

To examine whether low-temperature treatments of varying durations affect floral bud development in chrysanthemums, the opening status of inflorescences of ‘Xiaokuixiang’ was observed. Comparison of fully opened individual floral buds across treatment groups showed that the number of normally opened floral buds increased with longer low-temperature treatment duration, while the inflorescence diameter decreased correspondingly (**Figures 7B, C**).

### 3.8 Expression analysis of low-temperature vernalization-related genes during cold treatment

To investigate the regulatory role of low-temperature on floral induction at the gene level, the expression patterns of key genes during cold treatment and subsequent recovery at normal temperature were analyzed in this study (**Figure 7D**). As a core regulator of the vernalization pathway, the *CmFLC* gene exhibited a significant response to low-temperature stress. The results showed that *CmFLC* expression was significantly up-regulated during cold treatment compared to the control. However, its expression was sharply down-regulated after one week of recovery at normal temperature (LT5 + 1 w) and continued to decrease to a lower level by five weeks post-recovery (LT5 + 5 w). This trend indicates that *CmFLC* expression is induced by low-temperature but shows a pronounced downregulation trend during subsequent development at normal temperature, which may be closely related to the release of floral inhibition and the initiation of reproductive growth.

Furthermore, the expression of *CmGA20ox1*, a key enzyme gene in Gibberellin (GA) biosynthesis, was also significantly regulated by temperature changes. Its expression level was significantly lower than that of the control group during cold treatment. However, a significant recovery in *CmGA20ox1* expression was observed after one week of recovery at normal temperature (LT5 + 1 w), which is consistent with previous findings from our research group regarding low-temperature-regulated flowering in chrysanthemum (**Figure 7D**).

## 4 Discussion

Photoperiod is a core environmental signal regulating the transition from vegetative growth to reproductive growth in plants. This study confirmed that ‘Xiaokuixiang’ exhibits typical Facultative short-day plant (FSP) characteristics, with the most significant floral induction occurring under an 8 h light/16 h dark photoperiod (**Figures 3A, B**). This finding is consistent with previous research on autumn chrysanthemums (Yang et al., 2014; Higuchi et al., 2013; Higuchi and Hisamatsu, 2015). However, ‘Xiaokuixiang’ is capable of initiating flowering under day-neutral conditions, though with a low flowering rate, and fails to flower entirely under long-day conditions. Nevertheless, after reaching a certain stage of vegetative growth, it can differentiate flower buds at the apical meristem; these buds will cease development if they remain under long-day conditions. This indicates that its floral bud differentiation is dependent on short days, but not strictly obligate to them (Walters et al., 2004).

Increasing light intensity to 300 μmol m^-^² s^-^¹ significantly shortened the vegetative period and promoted early flowering in ‘Xiaokuixiang’ (**Figure 1**). This aligns with findings in Gerbera, where high light enhances carbon assimilation to support bud differentiation (Llewellyn et al., 2020). Mechanistically, high light intensity likely accelerates flowering by promoting continuous gibberellin (GA) synthesis in apical tissues. This is supported by the upregulation of *CmGA20ox1* in apical buds (**Figures 2A**, **5A**), suggesting GA mediates light intensity signals to drive the floral transition (Tang et al., 2025). Regarding temperature sensitivity, ‘Xiaokuixiang’ exhibits a dual response to photoperiod and temperature. While low-temperature treatment had a minor effect on budding time, it significantly optimized plant architecture and increased the number of normally opened buds (**Figure 7**). For this tea chrysanthemum, such induction boosts both ornamental value and yield, consistent with theories on low-temperature promoting floral differentiation (Yang and Cai, 2008). Molecularly, *CmFLC* was highly expressed during the 4 °C treatment but sharply downregulated after vernalization (LT5 + 1w). This relief of repression coincided with the recovery of *CmGA20ox1* expression (**Figure 7D**), indicating that GA biosynthesis was released from *FLC*-mediated repression. This confirms our team’s previous model that *FLC* homologs regulate flowering by modulating GA synthesis (Lyu et al., 2022), aligning with broader *FLC* expression patterns (Tao et al., 2017; Whittaker and Dean, 2017; Wang et al., 2022).

Furthermore, ‘Xiaokuixiang’ integrates these environmental signals through complex gene networks. We observed that *CmFTL3* was significantly upregulated under short-day and high-light conditions, mirroring the timing of bud emergence (**Figures 2C**, **5C**). This suggests a regulatory mechanism similar to *C. seticuspe*, where *CsFTL3* induces floral identity genes under short days (Nakano et al., 2019). The pivotal role of *FTL3* in mediating photoperiodic responses—characterized by decreased *AFT* and increased *FTL3* expression under critical photoperiods—appears conserved across chrysanthemum ecotypes (Takahashi et al., 2025). Additionally, short-day conditions likely accelerate the juvenile-to-adult transition by enhancing endogenous GA content and responsiveness (Zhao et al., 2023), involving complex signaling networks such as *CmGAST1*-mediated GA/calcium integration and the circadian clock gene *CmLHY* (Liu et al., 2025; Yuan et al., 2025).

It is noteworthy that in the Developmentally arrested flower buds observed in this study, the expression levels of key floral organ development genes such as *CmLFY*, *CmAFL1*, and *CmSEP3* were significantly lower than those in normal flower buds (**Figure 4B**). This elucidates the molecular basis underlying arrested floral bud development in response to the inhibition of apical dominance under long-day conditions, a phenomenon triggered by prolonged photoperiods or unfavorable environments (Okada, 1949; Whittaker and Dean, 2017). In Arabidopsis thaliana, *LFY* is specifically expressed in floral meristems (FMs) (Weigel et al., 1992). Through interaction with *UFO* and *SEPALLATA3* (*SEP3*), *LFY* activates floral organ identity genes to initiate flower development (Chae et al., 2008; Pelaz et al., 2000). *SEP3* is a core class E gene determining floral organ identity, responsible for integrating class A, B, and C proteins to form functional complexes (Pelaz et al., 2000). When ‘Xiaokuixiang’ is under a non-inductive photoperiod (long-day), the shoot apical meristem (SAM) initiates preliminary differentiation morphologically. However, due to the deficiency of upstream signals in the flowering pathway, downstream genes such as *CmSEP3* cannot reach the expression level required to trigger complete floral organ development. The malformed buds represent a unique physiological state of ‘initiation without completion’. Unlike normal floral arrest, this phenomenon is characterized by morphological initiation but molecular insufficiency, specifically the low expression of ABCE model genes under LD conditions. The results of this study clearly demonstrate that photoperiod not only controls the “timing” of flowering, but also determines the completeness of floral organ development by regulating the expression levels of ABCE model genes.

Comprehensive analysis indicates that precise flowering time control of ‘Xiaokuixiang’ should take into account the synergistic effects of light, temperature, water and nutrients. This study concludes that plants that have undergone winter low-temperature induction can achieve the fastest and highest-quality flowering under the combined conditions of relatively strong light (300 μmol m^-^² s^-^¹) and short-day (8 hours of light/16 hours of dark).

In practical production, the technical pathway proposed in this study can be adopted: select plants that have experienced natural low-temperature for cuttings, and implement shading treatment in a timely manner when vegetative growth reaches a certain stage (e.g., plant height of 28 cm). This precise regulation model can not only address the issues of high production costs and difficult quality control in the industrialization of fragrant chrysanthemum but also ensure the year-round stable supply of high-quality ‘Xiaokuixiang’ tea chrysanthemum.

## 5 Conclusions

This study established a precise regulatory protocol for the ‘Xiaokuixiang’, identifying photoperiod as the primary determinant of flowering and temperature as a critical modulator. In practical cultivation, maintaining a strict 8-h light/16-h dark photoperiod is essential to effectively prevent flower bud abortion and the formation of malformed buds, thereby ensuring high-quality commercial characteristics. We demonstrated that increasing light intensity to 300 μmol m^-^² s^-^¹ significantly accelerates plant development, providing a viable strategy to shorten the cultivation cycle. Furthermore, the introduction of winter low-temperature vernalization was found to optimize plant architecture and increase flower bud numbers by alleviating floral repression. Thus, the proposed integrated protocol—combining winter vernalization with high-light-intensity short-day induction—offers a standardized solution for achieving year-round production, enhancing flower quality, and improving overall production efficiency of aromatic chrysanthemums.

## Data Availability

The raw data supporting the conclusions of this article will be made available by the authors, without undue reservation.

## References

[B1] Andrés F. Coupland G. (2012). The genetic basis of flowering responses to seasonal cues. Nat. Rev. Genet. 13, 627–639. doi: 10.1038/nrg3291 22898651

[B2] Boss P. K. Bastow R. M. Mylne J. S. Dean C. (2004). Multiple pathways in the decision to flower: enabling, promoting, and resetting. Plant Cell 16, S18–S31. doi: 10.1105/tpc.015958 15037730 PMC2643402

[B3] Chae E. Tan Q. K.-G. Hill T. A. Irish V. F. (2008). An Arabidopsis F-Box protein acts as a transcriptional co-factor to regulate floral development. Development 135, 1235–1245. doi: 10.1242/dev.015842 18287201

[B4] Czechowski T. Stitt M. Altmann T. Udvardi M. K. Scheible W. R . (2005). Genome-wide identification and testing of superior reference genes for transcript normalization in Arabidopsis. Plant Physiol. 139, 5–17. doi: 10.1104/pp.105.063743 16166256 PMC1203353

[B5] Higuchi Y. Hisamatsu T. (2015). CsTFL1, a constitutive local repressor of flowering, modulates floral initiation by antagonizing florigen complex activity in chrysanthemum. Plant Sci. 237, 1–7. doi: 10.1016/j.plantsci.2015.04.011 26089146

[B6] Higuchi Y. Narumi T. Oda A. Nakano Y. Sumitomo K. Fukai S. . (2013). The gated induction system of a systemic floral inhibitor, antiflorigen, determines obligate short-day flowering in chrysanthemums. Proc. Natl. Acad. Sci. U.S.A. 110, 17137–17142. doi: 10.1073/pnas.1307617110 24082137 PMC3801008

[B7] Hopper D. A. Hammer P. A. (1991). Regression models describing Rosa hybrida response to day/night temperature and photosynthetic photon flux. J. Am. Soc Hortic. Sci. 116, 609–617. doi: 10.21273/jashs.116.4.609

[B8] Huang L. N. (2014). Some physiological responses of Phalaenopsis to different light intensities. Fujian Agriculture and Forestry University, Fuzhou. Master’s thesis.

[B9] Huang H. Wang Z. Zhang G. Liu Y. Wen J. Zheng Y. . (2026). Synergistic regulation by the stay-green gene family maintains green flower coloration via chlorophyll degradation in chrysanthemum. Plant Physiol. Biochem. 235, 111388. doi: 10.1016/j.plaphy.2026.111388 42142444

[B10] Kobayashi Y. Weigel D. (2007). Move on up, it's time for change-mobile signals controlling photperiod-dependent flowering. Genes Dev. 21, 2371–2384. doi: 10.1101/gad.1589007 17908925

[B11] Liu W. Li J. Zhang H. Wang Z. Aiwaili P. Yuan Y. . (2025). The peptide CmGAST1 integrates calcium and gibberellin signaling to regulate flowering in chrysanthemum. Plant Cell. 37(11), koaf269. doi: 10.1093/plcell/koaf269 41219161

[B12] Liu D. Zhan J. Wang S. Chen L. Zhu Q. Nie R. . (2024). Chrysanthemum morifolium attenuates metabolic and alcohol-associated liver disease via gut microbiota and PPARα/γ activation. Phytomedicine 130, 155774. doi: 10.1016/j.phymed.2024.155774 38820659

[B13] Livak K. J. Schmittgen T. D. (2001). Analysis of relative gene expression data using real-time quantitative PCR and the 2^(-Delta Delta C(T)) method. Methods 25, 402–408. doi: 10.1006/meth.2001.1262 11846609

[B14] Llewellyn D. Schiestel K. Zheng Y. (2020). Increasing levels of supplemental LED light enhances the rate flower development of greenhouse-grown cut gerbera but does not affect flower size and quality. Agronomy 10, 1332. doi: 10.3390/agronomy10091332 30654563

[B15] Lu S. Y. Yang Z. Q. (2021). Regulation of photoperiod on growth and flowering of cut chrysanthemum. Chin. J. Agrometeorol. 42, 596–605. doi: 10.3969/j.issn.1000-6362.2021.07.006

[B16] Lyu J. Aiwaili P. Gu Z. Xu Y. Zhang Y. Wang Z. . (2022). Chrysanthemum MAF2 regulates flowering by repressing gibberellin biosynthesis in response to low-temperature. Plant J. 112, 1159–1175. doi: 10.1111/tpj.16002 36214418 PMC10092002

[B17] Mouradov A. Cremer F. Coupland G. (2002). Control of flowering time: interacting pathways as a basis for diversity. Plant Cell 14, S111–S130. doi: 10.1105/tpc.001362 12045273 PMC151251

[B18] Nakano Y. Takase T. Takahashi S. Sumitomo K. Higuchi Y. Hisamatsu T. (2019). Chrysanthemum requires short-day repeats for anthesis: Gradual CsFTL3 induction through a feedback loop under short-day conditions. Plant Sci. 283, 247–255. doi: 10.1016/j.plantsci.2019.01.023 31128695

[B19] Okada M. (1949). Studies on crown bud of Chrysanthemum (1). J. Jpn. Soc Hortic. Sci. 18, 226–232. doi: 10.2503/jjshs.18.226

[B20] Pelaz S. Ditta G. S. Baumann E. Wisman E. Yanofsky M. F . (2000). B and C floral organ identity functions require SEPALLATA MADS-box genes. Nature 405, 200–203. doi: 10.1038/35012103 10821278

[B21] Shu F. Sarsaiya S. Ren L. Jin L. Hu Y. Qiao L. . (2024). Metabolomic analysis of plant-derived nanovesicles and extracellular vesicles from Pinellia ternata: insights into a temporary immersion bioreactor system. Physiol. Plant 176, e70016. doi: 10.1111/ppl.70016 39703077

[B22] Shu F. X. Yang N. X. Yu F. L. Luo P. Chen G. G. Chen J. S. (2025). Temporary immersion bioreactor system: an excellent strategy for preparing high-quality controlled extracellular vesicles from food and medicine homology plants. Food. Med. Homol. 2, 9420123. doi: 10.26599/FMH.2025.9420123

[B23] Takahashi S. Nakano Y. Sumitomo K. Hisamatsu T. Oda A. Onoue N. . (2025). Photoperiodic flowering and AFT/FTL3 gene expression in flowering-time varieties in chrysanthemum. Physiol. Plant 177, e70086. doi: 10.1111/ppl.70086 39887355

[B24] Tang J. Liu P. Yu Q. Wang H. Zhang X. Peng J. . (2025). The CmDOF6 transcription factor controls chrysanthemum plant height by repressing CmGA20ox1 via CmTCP8. Plant Physiol. 199(3), kiaf509. doi: 10.1093/plphys/kiaf509 41092130

[B25] Tao Z. Shen L. Gu X. Wang Y. Yu H. He Y. (2017). Embryonic epigenetic reprogramming by a pioneer transcription factor in plants. Nature 551, 124–128. doi: 10.1038/nature24300 29072296

[B26] Torres A. P. Lopez R. G. (2011). Photosynthetic daily light integral during propagation of Tecoma stans influences seedling rooting and growth. HortScience 46, 282–286. doi: 10.21273/hortsci.46.2.282

[B27] Walters R. G. Ibrahim D. G. Horton P. Kruger N. J. (2004). A mutant of Arabidopsis lacking the triose-phosphate/phosphate translocator reveals metabolic regulation of starch breakdown in the light. Plant Physiol. 135, 891–906. doi: 10.1104/pp.104.040469 15173568 PMC514124

[B28] Wang Q. Wang L. Cheng H. Wang S. Li J. Zhang D. . (2024). Two B-box proteins orchestrate vegetative and reproductive growth in summer chrysanthemum. Plant Cell Environ. 47, 2923–2935. doi: 10.1111/pce.14919 38629334

[B29] Wang X. Yao Y. Wen S. Bin J. Tan Q. Lou J. . (2022). Genome-wide characterization of Chrysanthemum indicum nuclear factor Y, subunit C gene family reveals the roles of CiNF-YCs in flowering regulation. Int. J. Mol. Sci. 23, 12812. doi: 10.3390/ijms232112812 36361603 PMC9654237

[B30] Wang Z. Yuan Y. Hong B. Zhao X. Gu Z . (2021). Characteristic volatile fingerprints of four chrysanthemum teas determined by HS-GC-IMS. Molecules 26, 7113. doi: 10.3390/molecules26237113 34885694 PMC8658894

[B31] Weigel D. Alvarez J. Smyth D. R. Yanofsky M. F. Meyerowitz E. M . (1992). LEAFY controls floral meristem identity in Arabidopsis. Cell. 69, 843–859. doi: 10.1016/0092-8674(92)90295-n 1350515

[B32] Whittaker C. Dean C. (2017). The FLC locus: a platform for discoveries in epigenetics and adaptation. Annu. Rev. Cell Dev. Biol. 33, 555–575. doi: 10.1146/annurev-cellbio-100616-060546 28693387

[B33] Xu S. Chong K. (2018). Remembering winter through vernalisation. Nat. Plants 4, 997–1009. doi: 10.1038/s41477-018-0301-z 30478363

[B34] Yamaguchi A. Abe M. (2012). Regulation of reproductive development by non-coding RNA in Arabidopsis: to flower or not to flower. J. Plant Res. 125, 693–704. doi: 10.1007/s10265-012-0513-7 22836383 PMC3485539

[B35] Yang Z. H. Cai H. (2008). Effects of low-temperature treatment at seedling stage on flowering of Tagetes patula. Anhui. Agric. Sci. Bull. 2008(14), 118–119. doi: 10.16377/j.cnki.issn1007-7731.2008.14.054

[B36] Yang Y. Ma C. Xu Y. Wei Q. Imtiaz M. Lan H. . (2014). A zinc finger protein regulates flowering time and abiotic stress tolerance in chrysanthemum by modulating gibberellin biosynthesis. Plant Cell 26, 2038–2054. doi: 10.1105/tpc.114.124867 24858937 PMC4079367

[B37] Yuan Y. Wang Z. Dong R. Gu Z. Xu Y. Gao J. . (2025). LATE ELONGATED HYPOCOTYL is a core component of far-red light-induced flowering in chrysanthemum. Hortic. Plant J. S2468-0141(25), 00147–5. doi: 10.1016/j.hpj.2025.04.010 38826717

[B38] Zhao X. Liu W. Aiwaili P. Zhang H. Xu Y. Gu Z. . (2023). PHOTOLYASE/BLUE LIGHT RECEPTOR2 regulates chrysanthemum flowering by compensating for gibberellin perception. Plant Physiol. 193, 2848–2864. doi: 10.1093/plphys/kiad503 37723123 PMC10663108

[B39] Zhou Q. Zhang S. S. Bao M. Z. Liu G. F. (2018). Research progress on molecular mechanisms of flowering induction in higher plants. Mol. Plant Breed. 16, 3681–3692. doi: 10.13271/j.mpb.016.003681

