# Supplementary material for: Photoperiod, light intensity, and vernalization regulate flowering in the novel fragrant Chrysanthemum ‘Xiaokuixiang’
Source: Front Plant Sci. 2026 Jul 1;17:1865852. doi: 10.3389/fpls.2026.1865852 (PMC13369048; doi:10.3389/fpls.2026.1865852)
Supplement: Supplementary file 1 [file Table1.docx]

Supplementary Material

# Supplementary Figures and Tables

##
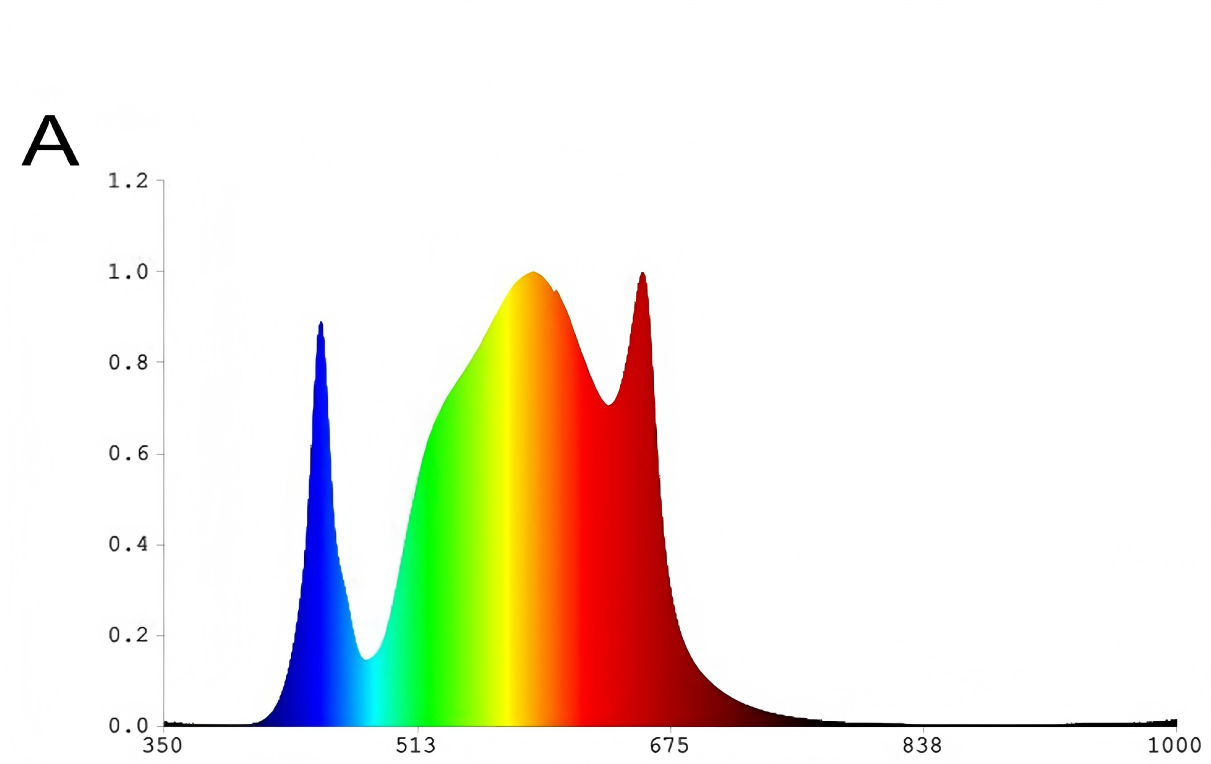
Supplementary Figures

**Supplementary Figure 1.** The spectral distribution of full-spectrum LED lamps

## Supplementary Table

| Table S1. Primers for qRT-PCR | | |
| --- | --- | --- |
| **Gene Symbol** | **Forward primer** | **Reverse Primer** |
| **CmUBI** | AGCTGAGCAGACTCCCGATG | AGGCGAATCATCAGTACCAAGT |
| **CmGA20ox** | TGTGGACAATGAGTGGCGTT | TGTCAGCCCTGTAATGCTTCTG |
| **CmCOL1** | CCTCTTCCTCATCCTCCTCCTCATC | CGTCATTCCAGAACTCTCCTTTGCC |
| **CmFTL3** | GGGAAAGTGGATTTGGTGGACG | GTCTTACAATTTGGTACTGTCG |
| **CmSOC1** | AACATGCTTCTTAGGTTTCAAGTG | TTAGCTGAGATGGGGATGCTT |
| **CmFLC** | AGACGGTGGATAGGCTTGTGG | TTGCTGATGCGACCTGTTG |
| **CmAFL1** | ATGCCGATGTTGGACTCGTT | GTTGCGGTAAGCTGCCTTTC |
| **CmLFY** | CTATCGCGGCTAGACAAGGTT | ACCACCAGCATCGTTACCAC |
| **CmSEP3** | AAACAGGCAGGTGACTTTTGC | ATTGGCTGATCGGCTGACTT |
